# Pretreatment multiparametric MRI radiomics-integrated clinical hematological biomarkers can predict early rapid metastasis in patients with nasopharyngeal carcinoma

**DOI:** 10.1186/s12885-024-12209-6

**Published:** 2024-04-08

**Authors:** Xiujuan Cao, Xiaowen Wang, Jian Song, Ya Su, Lizhen Wang, Yong Yin

**Affiliations:** 1 Shandong University Cancer Center, Shandong University, Jinan, Shandong China; 2 Department of Radiation Oncology, Shandong Cancer Hospital and Institute, Shandong First Medical University and Shandong Academy of Medical Sciences, Jinan, Shandong China; 3 Medical Imageology, Shandong Medical College, Jinan, China; 4 Department of Radiation Oncology Physics and Technology, Shandong Cancer Hospital and Institute, Shandong First Medical University and Shandong Academy of Medical Sciences, Jiyan Road 440, Jinan, Shandong 250117 People’s Republic of China

**Keywords:** Nasopharyngeal carcinoma, Magnetic resonance imaging, Radiomics, Rapid metastasis, Prediction model

## Abstract

**Background:**

To establish and validate a predictive model combining pretreatment multiparametric MRI-based radiomic signatures and clinical characteristics for the risk evaluation of early rapid metastasis in nasopharyngeal carcinoma (NPC) patients.

**Methods:**

The cutoff time was used to randomly assign 219 consecutive patients who underwent chemoradiation treatment to the training group (*n* = 154) or the validation group (*n* = 65). Pretreatment multiparametric magnetic resonance (MR) images of individuals with NPC were employed to extract 428 radiomic features. LASSO regression analysis was used to select radiomic features related to early rapid metastasis and develop the Rad-score. Blood indicators were collected within 1 week of pretreatment. To identify independent risk variables for early rapid metastasis, univariate and multivariate logistic regression analyses were employed. Finally, multivariate logistic regression analysis was applied to construct a radiomics and clinical prediction nomogram that integrated radiomic features and clinical and blood inflammatory predictors.

**Results:**

The NLR, T classification and N classification were found to be independent risk indicators for early rapid metastasis by multivariate logistic regression analysis. Twelve features associated with early rapid metastasis were selected by LASSO regression analysis, and the Rad-score was calculated. The AUC of the Rad-score was 0.773. Finally, we constructed and validated a prediction model in combination with the NLR, T classification, N classification and Rad-score. The area under the curve (AUC) was 0.936 (95% confidence interval (95% CI): 0.901–0.971), and in the validation cohort, the AUC was 0.796 (95% CI: 0.686–0.905).

**Conclusions:**

A predictive model that integrates the NLR, T classification, N classification and MR-based radiomics for distinguishing early rapid metastasis may serve as a clinical risk stratification tool for effectively guiding individual management.

**Supplementary Information:**

The online version contains supplementary material available at 10.1186/s12885-024-12209-6.

## Introduction

Nasopharyngeal carcinoma (NPC) has the highest incidence among malignant tumors of the head and neck [1–3]. Radiotherapy plays a fundamental role in the treatment of nasopharyngeal carcinoma (NPC) due to its remarkable degree of radiosensitivity. The occurrence of local recurrence and/or distant metastases constitutes the primary cause of therapy failure in patients with this condition. The implementation of intensity-modulated radiation therapy and concomitant chemoradiotherapy has significantly enhanced the local control rate of NPC, leading to a remarkable 5-year overall survival (OS) rate of 80%-88%. However, it is important to note that distant metastasis has emerged as the predominant pattern of treatment failure, accounting for the majority of cases [4]. Approximately 10% of individuals with NPC present with distant metastasis upon initial diagnosis, while an additional 10–20% progress to metastasis following treatment, resulting in a 5-year survival rate below 10% [5]. Metastasis profoundly affects patients' quality of life and rapidly progresses, leading to mortality [6].

In clinical practice, the tumor-node-metastasis (TNM) classification system serves as a crucial tool for clinical decision-making and prognostic assessment in tumor patients, including those with nasopharyngeal carcinoma (NPC). Although patients with the same classification are typically treated with similar therapeutic approaches, their outcomes and prognoses may significantly differ. This discrepancy suggests that the TNM classification system primarily considers the relationship between the tumor and surrounding tissues and organs and fails to incorporate intratumor characteristics and heterogeneity, thereby limiting its ability to accurately predict risk stratification markers. In contrast, magnetic resonance imaging (MRI), with its superior soft-tissue resolution, offers more precise visualization of microscopic lesions and the exact extent of lesions [7]. Currently, MRI is widely employed for tumor staging, image guidance, and follow-up via manual visual interpretation. However, the clinical application of MRI is primarily focused on qualitative structural information. To overcome this limitation, a modern technology called 'radiomics' [8] can extract high-throughput quantitative information, enabling its use in clinical diagnosis, prognosis, and treatment evaluation. Numerous studies across various cancer types, including head and neck squamous cell carcinoma [9–11], lung cancer [12], rectal cancer [13, 14], and breast cancer [15, 16], have demonstrated the prognostic value of radiomics. These studies have linked radiomics to local recurrence, pathological molecular classification, and pathological remission after neoadjuvant chemotherapy [17]. Additionally, the formation of oxygen-free radicals resulting from persistent oxidative stress and the inflammatory response is directly associated with the onset and progression of cancer from a pathophysiological perspective [18]. Thus, inexpensive and easily identifiable inflammatory blood markers, such as the lymphocyte-to-monocyte ratio (LMR), platelet-to-lymphocyte ratio (PLR), neutrophil-to-lymphocyte ratio (NLR), and systemic immune-inflammation index (SII), have emerged as potential prognostic indicators for NPC [19, 20]. Consequently, there is a crucial need to explore biomarkers accurately from multiple dimensions. This exploration could facilitate the development of more aggressive and personalized treatment plans for high-risk patients in a timely manner. To address this need, we constructed and validated a combined model aimed at early prognostication of the likelihood of rapid metastasis.

## Methods

### Patients

**Fig. 1:**
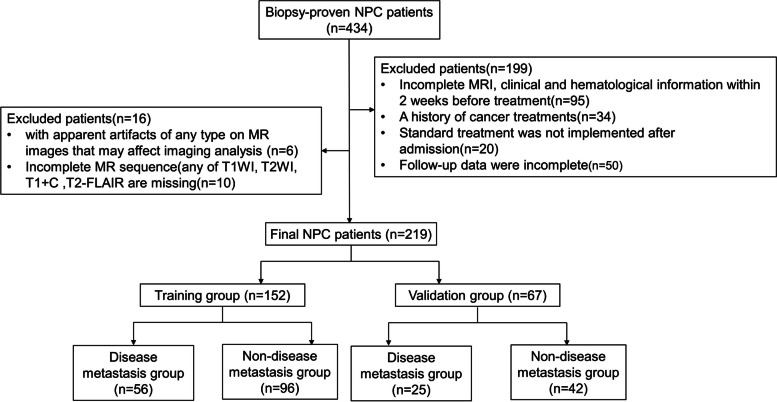
Flow chart for patient inclusion

A retrospective study was conducted with 434 consecutive patients who had NPC with a histological diagnosis and who were treated at the Shandong Cancer Hospital ana Institute between July 2016 and December 2022. The inclusion criteria were as follows: (1) underwent a nasopharynx-neck MRI within two weeks before any antitumor medication, without any obvious artifacts of any form on MR images that would influence imaging analysis; and (2) complete baseline clinical and hematological records. (3) Patients with nasopharyngeal carcinoma without metastasis at first diagnosis. The exclusion criteria included any of the following: (1) incomplete MRI, clinical or hematological information within 2 weeks before treatment; (2) a second tumor combined with distant metastasis before cancer treatment; (3) no complete standard treatment; or (4) incomplete follow-up data. In total, 219 consecutive NPC patients who met the inclusion criteria were included and are illustrated in Fig. 1. Patients were randomly divided into a training group (152 patients) and a validation group (67 patients) according to the cutoff date of July 31, 2020. The training cohort was used for the development of the prognostic model, and the generalizability of the model was evaluated using the validation cohort. During the 24-month follow-up period, patients with NPC were divided into two groups: those with distant metastasis were placed in the disease metastasis group, while those without distant metastasis were placed in the non-disease metastasis group. The Shandong Institute of Cancer Prevention and Treatment in China approved this study.

### Hematological examination

Hematology samples were collected from the study participants one week prior to their therapy. Blood samples were obtained from fasted individuals who had abstained from food for a period of 8–12 h. Blood was drawn into an anticoagulant vacuum tube and promptly analyzed within 30 min. The automated hematology analyzer Sysmex XT-1800i (Sysmex, Kobe, Japan) was used to analyze routine peripheral blood cells. The study included the lymphocyte-to-monocyte ratio (LMR), platelet-to-lymphocyte ratio (PLR), neutrophil-to-lymphocyte ratio (NLR), and systemic immune inflammation index (SII). The calculation formula was as follows: LMR = lymphocyte (10^9^/L)/serum albumin (g/L); PLR = platelet count (10^9^/L)/lymphocyte count (10^9^/L); NLR = neutrophil count (10^9^/L)/lymphocyte count (10^9^/L); and SII = platelet count (10^9^/L) *neutrophil count/lymphocyte count (10^9^/L).

### Treatment strategies and follow-up

All patients enrolled in the study received individualized treatment according to the guidelines established by the National Comprehensive Cancer Network (NCCN). Qualified patients underwent radiation treatment using intensity-modulated radiation therapy (IMRT). The gross tumor volumes were determined based on pretreatment assessments, including MRI, CT, positron emission tomography-computed tomography (PET-CT) scans, and nasopharynx laryngoscopy. Each patient received radiotherapy, with a therapeutic radiotherapy dose of 66–70 Gy divided into 33–35 fractions and IMRT once a day, 5 times a week for a total of 7 weeks. All patients received platinum-based concurrent chemoradiotherapy with a dose of cisplatin of 80–100 mg/m^2^/3 weeks. Induction or adjuvant chemotherapy, including cisplatin (80 mg/m^2^) + 5-fluorouracil (1000 mg/m^2^), cisplatin (80 mg/m^2^) + gemcitabine (1000 mg/m^2^), and cisplatin (60 mg/m^2^) + docetaxel (60 mg/m^2^) + 5-fluorouracil (600 mg/m^2^), was repeated every 21 days for 2–3 cycles.

The diagnosis model of distant metastases was established through the evaluation of clinical symptoms and pertinent imaging findings, including nasopharyngeal-neck MRI, thoracic CT, whole-body bone scintigraphy, and PET-CT scans. To identify early metastases, a follow-up period of 24 months was selected as the cutoff, with the primary clinical objective being the detection of distant metastasis.

### MRI scan acquisition

All MR images were obtained using 3.0 T MR scanners with an 8-channel head and neck phased array coil (Achieva, Philips Medical Systems, Netherlands; Ingenia, Philips Medical Systems, Netherlands). Each patient's T1-weighted imaging (T1WI), contrast-enhanced T1-weighted imaging (CE-T1WI), T2-weighted imaging (T2WI), and T2-weighted imaging with fat saturation (T2WI/FS) images were obtained via the image archiving and communication system. The MRI sequences used were fast-spin echo (FSE) axial T1-weighted images (TR = 540 ms, TE = 11.8 ms), FSE axial T2-weighted images (TR = 4000 ms, TE = 99 ms), FSE axial T2-weighted images with fat saturation (TR = 8472 ms, TE = 85 ms), and contrast-enhanced FSE axial T1-weighted images (contrast agent = GdDTPA, Magnevist, Schering, Berlin, Germany; dose = 0.1 mmol/kg body weight; TR = 540 ms, TE = 11.8 ms). In all sequences, the slice thickness was 3 mm, with section gaps of 1 mm and a matrix of 512*512.

### Tumor segmentation and feature extraction

Tumor segmentation was performed by two radiologists (with 10 years of experience in head and neck imaging) utilizing Slicer 5.0.3, and then the two radiologists alternately verified the target area drawn. The ROIs were based on the T1WI, CE-T1WI, T2WI, and T2WI/FS images, so there was no need for image registration. Eighty ROIs of 20 patients were randomly selected from the target areas outlined by two radiotherapists for interrater reliability. The absolute agreement between radiomics characteristics gathered from the ROIs of eighty randomly selected individuals was then calculated using the intraclass correlation coefficient (ICC). The features with an ICC > 0.75 were considered strongly consistent.

PyRadiomics (http://www.radiomics.io/PyRadiomics.html) is a useful tool for extracting radiomic features from contoured ROIs [21]. The intensity range of the pixels was normalized from 0 to 100 because this study used MRI scanners with different field strengths. For each MRI scan, a total of 107 radiomics features were extracted (14 shape features, 18 first-order intensity statistics features, 14 Gy level dependence matrix features (GLDM), 24 Gy level cooccurrence matrix features (GLCM), 16 Gy level run length matrix features (GLRLM), 16 Gy level size zone matrix features (GLSZM), and 5 neighborhood gray tone difference matrix features (NGTDM)).

### Statistical analysis

Differences in clinical characteristics between the training and validation cohorts were compared with the chi-square test for categorical variables, and the Mann–Whitney U test was used to analyze the significance of differences in the nonnormally distributed individual parameters. The C-index was used to evaluate the predictive power of each model. ROC curve analysis was used to identify the best cutoff value for survival prediction. The continuous variable was transformed into a binary variable by the optimal cutoff value. R software 4.0.2 (http://www.r-project.org/) was used for a sizable portion of the statistical analyses. The R packages listed below were used: ICCs were calculated using the *irr* package (version 0.84.1); Least absolute shrinkage and selection operator (LASSO) regression was performed using the *glmnet* package (version 4.0–2); calibration curves and nomograms were generated using the *rms* package (version 6.0–1); and heatmaps were generated using the *pheatmap* (version 1.0.12) and *corrplot* (version 0.8) packages. A few of the statistical analyses, including univariate logistic regression and multivariate logistic regression, were performed using SPSS software (version 25). The area under the curves (AUCs) of the two models were compared using the DeLong test in MedCalc (version 20.009). Every statistical test was two-sided. Statistical significance was defined as a *p* value < 0.05.

## Results

### Patients

The data from July 31, 2020, were used to split the 219 patients into a training cohort (*n* = 152) and a validation cohort (*n* = 67). The training cohort had 36.8% of patients with distant metastases, and the validation cohort had 37.3%; no significant difference was identified between the two groups. The median duration of follow-up was 43.2 months (range: 1–79.1 months). The overall average progression-free survival (PFS) was 53.6 months, 51.7 months, and 47 months in the training and validation cohorts, respectively. The baseline clinicopathological features of the 219 eligible patients, including 81 patients with early rapid metastasis (37 with bone metastasis, 21 with liver metastasis, 12 with lung metastasis, 2 with brain metastasis, and 9 with multiple metastasis), are shown in Table 1. The differences in patient sex, age, TNM classification, clinical classification, EBV-DNA status, BMI, and BSA before treatment between the training and validation cohorts were well balanced (all *p* > 0.05).

**Table 1:** Patient characteristics of the training cohort and validation cohort

		**Training cohort (*n* = 152)**	**Validation cohort (*n* = 67)**	***p* value**
metastasis	no	96 (63.2%)	42 (62.7%)	1
	yes	56 (36.8%)	25 (37.3%)	
gender	female	37 (24.3%)	16 (23.9%)	1
	male	115 (75.7%)	51 (76.1%)	
age	< 55	89 (58.6%)	35 (52.2%)	0.471022503
	≥ 55	63 (41.4%)	32 (47.8%)	
PLR	< 260	134 (88.2%)	55 (82.1%)	0.322032508
	≥ 260	18 (11.8%)	12 (17.9%)	
NLR	< 3.8	107 (70.4%)	50 (74.6%)	0.632751819
	≥ 3.8	45 (29.6%)	17 (25.4%)	
LMR	< 5.5	129 (84.9%)	56 (83.6%)	0.968289603
	≥ 5.5	23 (15.1%)	11 (16.4%)	
SII	< 1185	113 (74.3%)	49 (73.1%)	0.983563436
	≥ 1185	39 (25.7%)	18 (26.9%)	
clinical classification	1–2	22 (14.5%)	10 (14.9%)	1
	3–4	130 (85.5%)	57 (85.1%)	
T classification	1–2	78 (51.3%)	30 (44.8%)	0.456065962
	3–4	74 (48.7%)	37 (55.2%)	
N classification	0, 1	89 (58.6%)	40 (61.5%)	0.681561
	2, 3	63 (41.4%)	25 (38.5%)	
EBv.DNA	< 400	120 (78.9%)	53 (79.1%)	1
	≥ 400	32 (21.1%)	14 (20.9%)	
BMI	< 28	131 (86.2%)	60 (89.6%)	0.63962412
	≥ 28	21 (13.8%)	7 (10.4%)	
BSA	< 1.5	11 (7.2%)	3 (4.5%)	0.558705859
	≥ 1.5	141 (92.8%)	64 (95.5%)	

*LMR* Lymphocyte-to-monocyte ratio, *PLR* Platelet-to-lymphocyte ratio, *NLR* Neutrophil-to-lymphocyte ratio, *SII* Systemic immune inflammation index, *EBv-DNA* Epstein–Barr virus DNA, *BMI* Body mass index, *BSA* Body surface area

### Clinical characteristics and hematological biomarker selection

We analyzed the relationships between clinical characteristics, hematological biomarkers and early rapid metastasis, and the details are shown in Table 2. We studied the importance of each variable via a logistic regression model. Univariate analysis indicated that the PLR, NLR, SII, clinical classification (III to IV), advanced T classification [3, 4] and N classification [3] were significantly associated with early rapid metastasis in the training cohort. According to the multivariate analysis, the NLR (*p* = 0.015), advanced T classification (*p* = 0.025) and N classification (*p* = 0.04) were found to be independent risk factors.

**Table 2:** Logistic regression of clinical and hematological characteristics

	**univariate logistic regression**				**multivariate logistics regression**			
	*P* value	Exp(B)	low	high	*P* value	Exp(B)	low	high
gender(male vs. female)	0.523	1.291	0.589	2.829				
age(< 55vs. ≥ 55)	0.342	1.382	0.709	2.692				
type	0.989							
type(1vs.3)	0.956	1.043	0.237	4.585				
type(2vs.3)	0.888	1.062	0.458	2.462				
PLR (< 260vs. ≥ 260)	0.002*	5.502	1.844	16.420	0.082	3.691	0.848	16.075
NLR(< 3.8vs. ≥ 3.8)	< 0.001*	4.034	1.938	8.398	0.015*	6.119	1.424	126.294
LMR(< 5.5vs. ≥ 5.5)	0.111	0.425	0.148	1.216				
SII(< 1185vs. ≥ 1185)	< 0.001*	4.050	1.887	8.694	0.586	0.648	0.136	3.083
clinical classification (I,IIvs.III,IV)	0.010*	7.105	1.594	31.679	0.096	4.472	0.767	26.070
T1,2 vs. 3,4	0.004*	2.747	1.388	5.438	0.025*	6.119	1.424	26.294
N0,1,2 vs. 3	0.015*	2.308	1.175	4.533	0.04*	3.305	1.466	7.454
EBV-DNA(-vs. +)	0.188	1.700	0.771	3.747				
BMI(< 28vs. ≥ 28)	0.539	1.340	0.526	3.415				
BSA(< 1.5vs. ≥ 1.5)	0.973	1.022	0.286	3.660				

*LMR* Lymphocyte-to-monocyte ratio, *PLR* Platelet-to-lymphocyte ratio, *NLR* Neutrophil-to-lymphocyte ratio, *SII* Systemic immune inflammation index, *EBv-DNA* Epstein–Barr virus DNA, *BMI* Body mass index, *BSA* Body surface area

^*^Indicates *p* < 0.05

### Radiomics feature extraction and feature selection

**Fig. 2:**
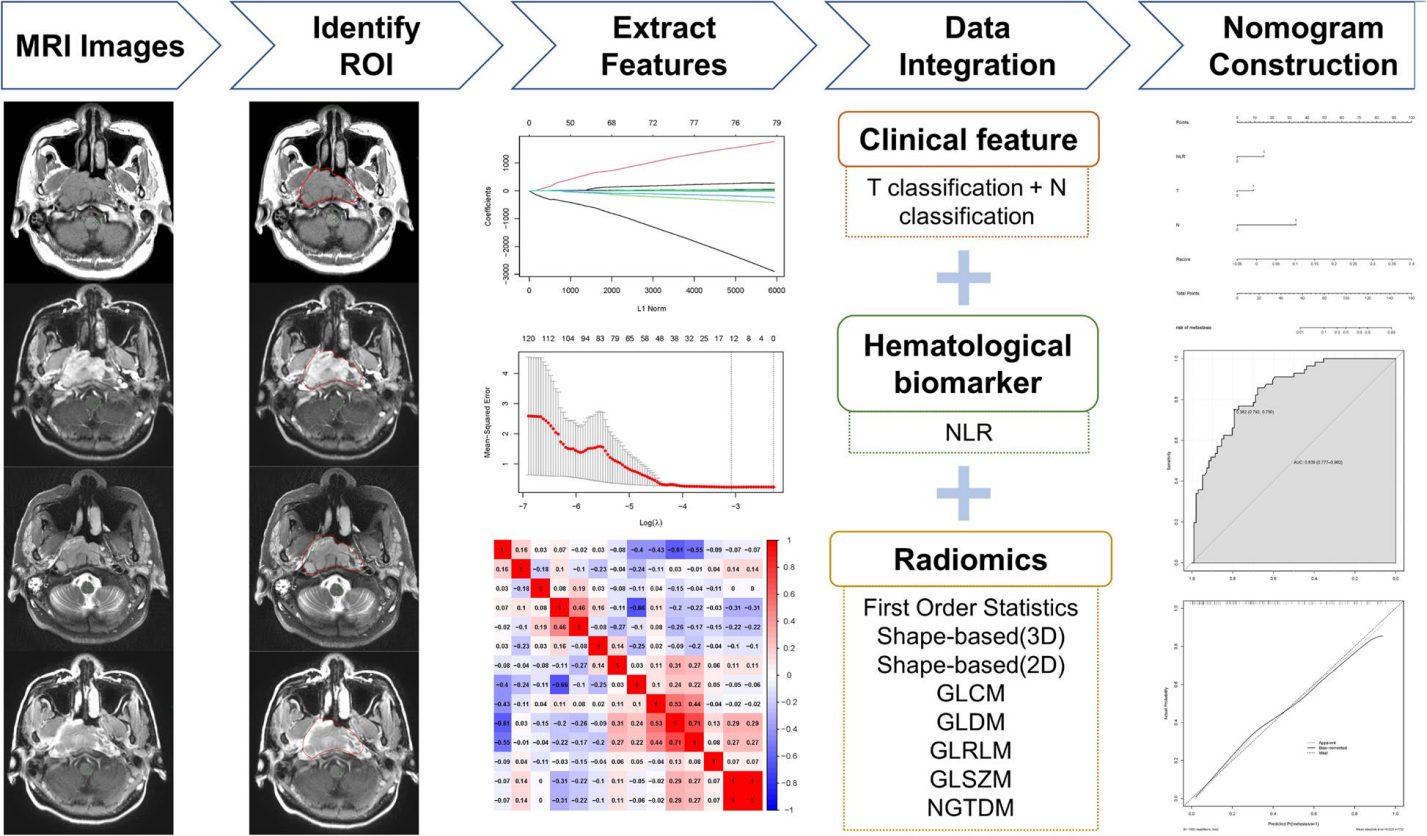
Flow chart for radiomic analysis

**Fig. 3:**
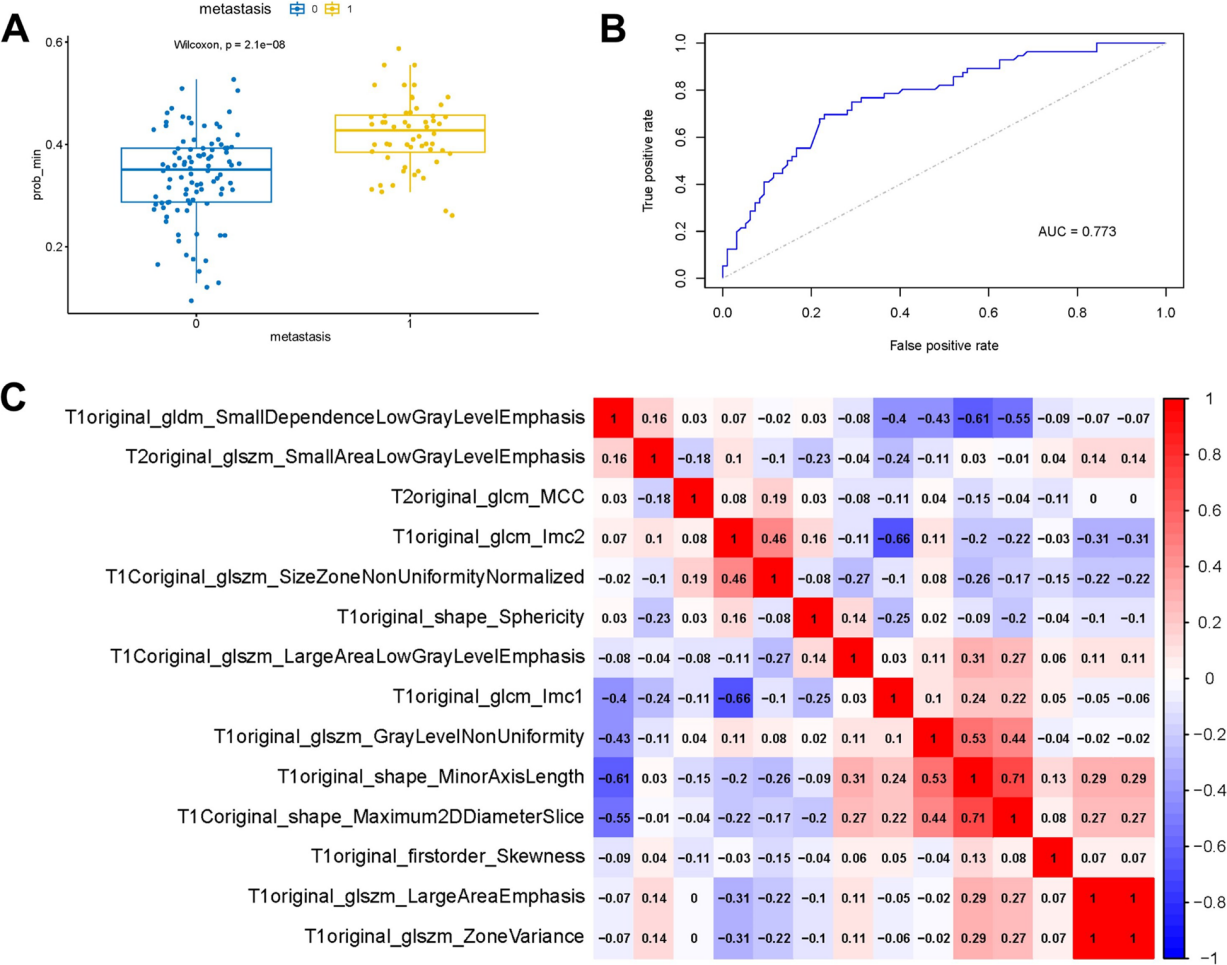
Fourteen radiomic features were selected by LASSO regression. **A** Analysis of differences in the Rad-score between the metastatic and non-metastatic groups (“0” indicates “metastasis”; “1” indicates “non-metastasis”) (*p* < 0.001). **B** The ROC curve of the radiomic model. **C** Pearson correlation coefficient of the 14 significant features

**Fig. 4:**
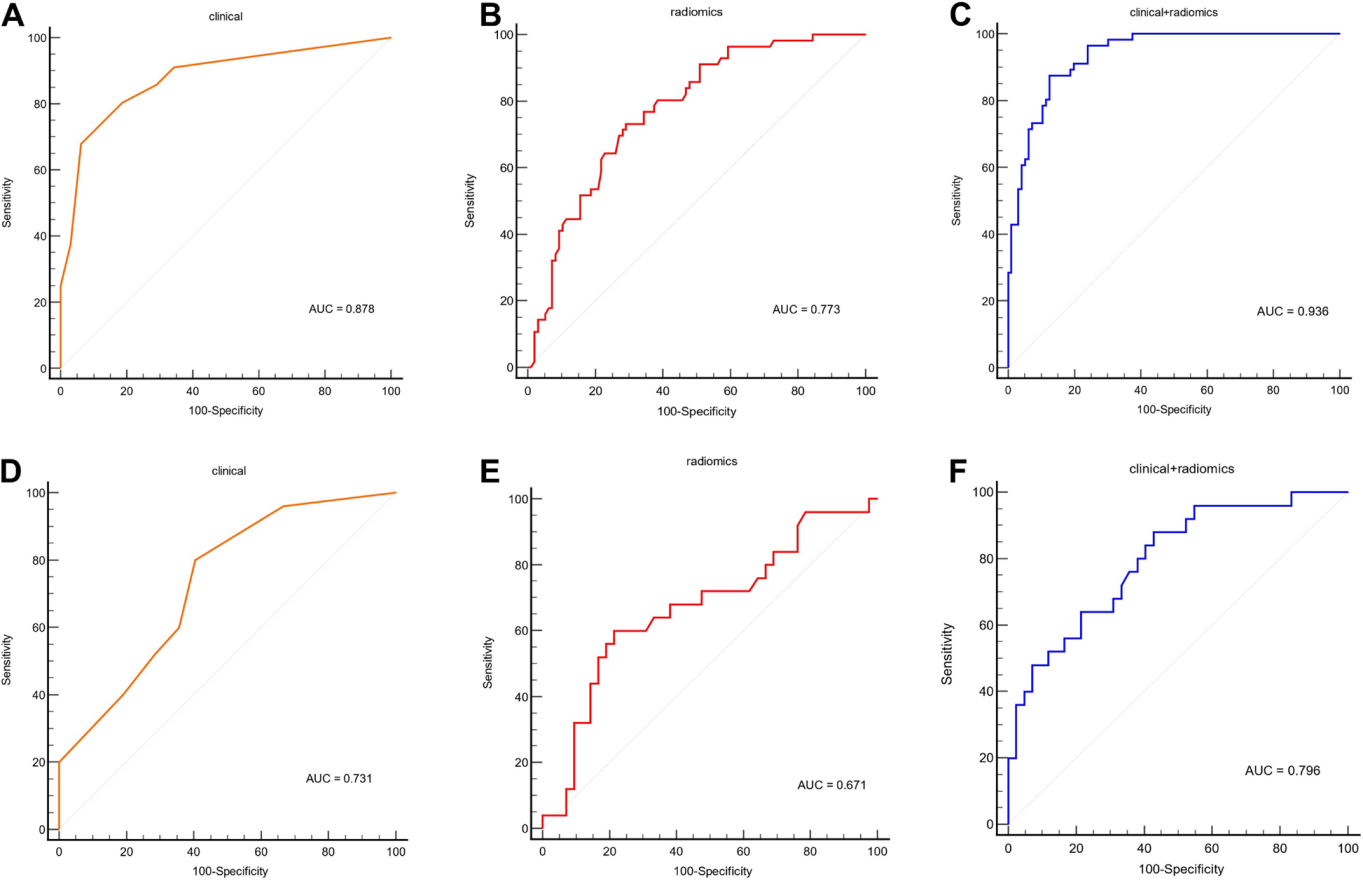
**A**, **D** ROC curve of the clinical prediction model consisting of the NLR, T classification and N classification in the training and validation cohorts. **B**, **E** ROC curve of the radiomic prediction model composed of the Rad-score in the training and validation cohorts. **C**, **F** ROC curves of the clinical + radiomic prediction model in the training and validation cohorts

An overview of the radiomic analysis process is presented in Fig. 2. On the training set, a total of 428 radiomic features were extracted from axial T1WI, CE-T1WI T2WI, and T2WI/FS images. We used LASSO regression analysis for dimension reduction. After tenfold cross-validation, we selected 14 features. The names of these features and their coefficients are shown in Table 3. We acquired the Rad-score using the coefficients of 14 characteristics and examined the difference in the Rad-score between the metastatic and nonmetastatic groups, and the two groups were significantly different (Fig. 3A). Then, we calculated the area under the curve (AUC) (0.773) (Fig. 3B). Pearson correlation analysis was used to evaluate the relationships between the 14 radiomic characteristics (Fig. 3C). Two features (T1original_glszm_ZoneVariance and T1Coriginal_shape_Maximum2DDiameterSlice) with |*r*| values greater than 0.7 were identified, and the AUC remained at 0.773 (Fig. 4B). We also separately analyzed the radiomic features of each of the four sequences (T1WI, CE-T1WI, T2WI, and T2WI/FS) of MRI pretreatment using LASSO regression. The AUCs for T1WI, CE-T1WI, T2WI, and T2WI/FS were 0.736, 0.715, 0.589, and 0.599, respectively (Supplementary Fig. 1).

**Table 3:** 14 radiomics characteristics related to metastasis

Texture type	Coefficient	Texture parameters
Shape	0.004861564	T1original_shape_MinorAxisLength
	-0.031214079	T1original_shape_Sphericity
	0.003104447	T1Coriginal_shape_Maximum2DDiameterSlice
GLSZM	3.66E-06	T1original_glszm_GrayLevelNonUniformity
	-1.10E-06	T1original_glszm_LargeAreaEmphasis
	-1.36E-08	T1original_glszm_ZoneVariance
	-0.001844463	T1Coriginal_glszm_LargeAreaLowGrayLevelEmphasis
	-0.24536114	T1Coriginal_glszm_SizeZoneNonUniformityNormalized
	0.135822198	T2original_glszm_SmallAreaLowGrayLevelEmphasis
GLDM	-3.24843171	T1original_gldm_SmallDependenceLowGrayLevelEmphasis
First order	0.037914991	T1original_firstorder_Skewness
GLCM	0.066257102	T1original_glcm_Imc1
	-0.201283621	T1original_glcm_Imc2
	-0.014619565	T2original_glcm_MCC
Intercept	0.423481837	

*Shape* Shape features, *First order* First-order intensity statistics features, *GLSZM* Gray level size zone matrix features, *GLCM* Gray level co-occurrence matrix features, *GLDM* Gray level dependence matrix features

### Establishment and comparison of predictive models

It can be concluded from the above that the NLR, advanced T classification and N classification were independent risk factors. We used the fitting formula obtained from the multivariate analysis of the NLR, T classification and N classification to construct a prediction model. The AUC of the clinical prediction model in the training dataset was 0.878 (Fig. 4A), and it was 0.731 in the validation dataset (Fig. 4D). We also learned from the above that multisequence MR radiomics can provide high prediction (training dataset: AUC = 0.773; validation dataset: AUC = 0.671) (Fig. 4B and E). To further increase the prediction accuracy, we created a model by integrating radiomics features with clinical hematological indices. We performed multivariate logistic regression analysis on the NLR, T classification, N classification and Rad-score and obtained the fitting formula for the total risk points: total points = 1.68*NLR + 1.0317*T-classification + 3.6911*N-classification + 24.35221*Rad-score-9.7082. The model's predictive capacity was assessed using ROC curve analysis. The AUC of the model for predicting early rapid metastasis reached 0.936 (CI: 0.901–0.971) in the training cohort (Fig. 4C) and 0.796 (CI: 0.686–0.905) in the validation cohort (Fig. 4F).

We compared the ROC curves of the three models in the training set and validation set and performed DeLong’s tests. In the training set, the combined model (consisting of clinical indicators and radiomics) showed better predictive performance than the clinical model and radiomic model (AUC: 0.936 vs. 0.878; *p* = 0.011 for Delong's test; AUC: 0.936 vs. 0.773; *p* = 0.001 for Delong's test) (Supplementary Table).

### Evaluation of nomogram prediction ability

**Fig. 5:**
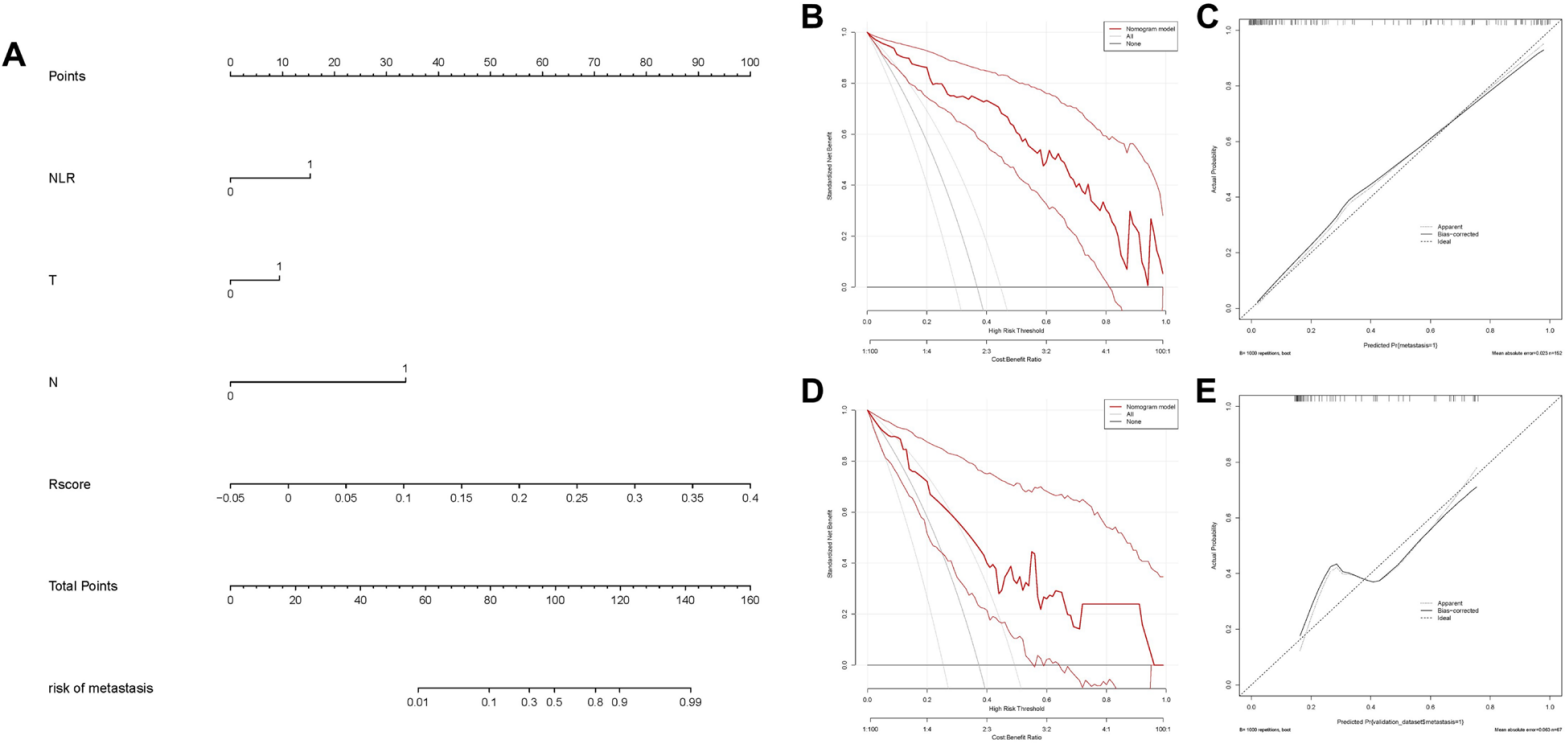
**A** A nomogram incorporating the NLR, T classification, N classification and Rad-score. **B**, **D** Calibration curves of the nomograms developed in the training and validation cohorts. **C**, **E** DCA curves of the nomograms developed in the training and validation cohorts

**Fig. 6:**
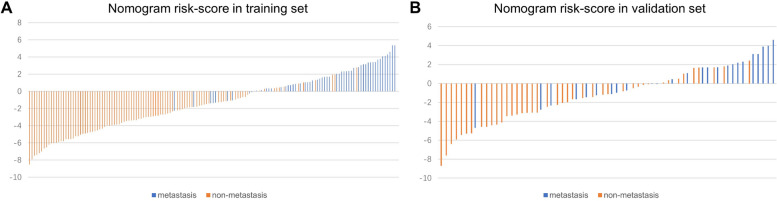
A bar chart of the nomogram’s risk score. **A** and **B** represent the training set and the validation set, respectively. The colors in the bar chart represent the real metastasis or non-metastasis groups, with orange representing the non-metastasis group and blue representing the metastasis group

We established a nomogram based on the combined model for visualization (Fig. 5A). The nomogram conveniently provided clinicians with a quantitative tool. The risk of metastasis can be determined using the calculated overall risk score. Decision curve analysis (DCA) was used to estimate the clinical utility of the models in clinical decision-making (Fig. 5B and D). A calibration plot (Fig. 5C and E) displayed how close the estimated risk from the nomogram was to the observed risk. The likelihood of disease progression was well calibrated in both the training and validation cohorts. The risk score is represented by a bar chart (Fig. 6A and B). The algorithm identified 58 individuals in the training cohort with potential metastases, 46 of whom experienced early rapid metastasis. In the validation set, the model predicted metastasis in 23 patients, 14 of whom developed early rapid metastasis. Therefore, we can conclude that, in addition to a small range of bias, patient intervention based on the predictive model can achieve greater clinical benefit.

## Discussion

In this study, we constructed a combined radiomic and clinical model based on radiomic features from baseline MR images before initiating treatment and clinical variables to predict the risk of early distant metastasis in NPC patients. The combined progression model showed superior predictive ability and was remarkably superior to the clinical model and radiomic model alone. This is the first study in which we extracted signatures of primary tumors from four-sequence MR images and evaluated the feasibility of predicting progression over two years by MR imaging-based radiomic features of local advanced nasopharyngeal carcinoma.

Because the effectiveness of radiotherapy and concurrent radiotherapy and chemotherapy significantly improved the local control rate of NPC, distant metastasis was the main failure mode. First, because approximately 59–73.5% of distant metastases occur within the first 2 years after complete remission of the nasopharynx tumor, early prediction of a high risk of disease progression after complete regression is vital and abstruse. This is an obstacle to adopting early intervention or more aggressive management for high-risk patients. Second, the treatment tolerance and reaction of patients with early distant metastasis are poor after simultaneous chemoradiotherapy, and the 5-year overall survival rate is less than 5% [22]. Therefore, it is necessary to predict the high risk of early metastasis in NPC patients before treatment to optimize individualized therapeutic strategies for early prevention [23–30]. *Chen* et al. reported the importance of predicting progression-free survival because early active intervention has been shown to improve mortality [1].

Due to the existence of tumor heterogeneity [31], it is crucial to find a method to more comprehensively and accurately capture microscopic characteristics and differences in tumors. We considered independent clinical factors and radiomics signatures to establish a composite model for predicting the risk of early distant metastasis. Radiomics signatures can reflect the pathophysiological information intrinsic to the tumor and provide abundant information. *Bao D* et al. evaluated the value of pretreatment MRI radiomics machine learning models in predicting disease progression in nasopharyngeal carcinoma patients who achieved a complete response after treatment and may help to improve clinical decision-making [28]. A previous study reported a machine learning model based on a combination of clinical and radiomic features in a developed cohort (AUC: 0.80) and a validated cohort (AUC: 0.80) that can discriminate 3-year disease progression equally well after primary treatment [31]. *Fang ZY* et al. reported that a clinical radiomic model exhibited superior prediction ability and accuracy compared to a simple clinical model or radiomic model alone [32, 33]. In another previous study, they developed a progressive radiomic model based on combined contrast-enhanced T1WI texture and TNM classification and achieved an AUC of 0.78, which was better than that of the TNM classification system alone for discriminating 3-year PFS (C-index: 0.761; 95% CI: 0.664 to 0.858), which was in accordance with our findings [34]. In our study, the AUC of our combined model for predicting early rapid metastasis reached 0.936 (95% CI: 0.901–0.971) in the training cohort and 0.796 (95% CI: 0.686–0.905) in the validation cohort, indicating that our model had better stability than the radiomic model alone.

Previous studies have tried their best to analyze the relationships between radiomics features and clinical outcomes, such as overall survival (OS) [35], local recurrence [24], and side effects after radiotherapy [36]. We focused on the risk of early distant metastasis and a worse prognosis. In contrast to studies that simply identified the lesion's largest cross-section, we constructed a 3D VOI from several successive slices of the whole tumor, which could more accurately represent the lesion's heterogeneity [37]. Tumor heterogeneity may be related to tumor angiogenesis, cell proliferation, necrosis, and even different tumor gene phenotypes [38]. Greater tumor heterogeneity is closely connected with poorer prognosis, which could be only associated with intrinsic aggressive biology or therapy resistance [39]. We extracted radiomics features from four sequences to construct a prognostic model that may be more comprehensive considering the characteristics of different MRI sequences and exhibited excellent performance for individual prediction [40]. *Wang* et al. reported that the radiomics model derived from multiple MR sequences had better predictive capability than that derived from a single MR sequence (*p* < 0.05) [41]. Moreover, the radiomics data extracted from functional sequences indicated invasive biological features of the tumor, leading to a high possibility of local disease progression. In our study, 12 radiomic features were retained, and these were the most important factors for predicting 2-year disease progression. First-order statistics describe the distribution of voxel intensities within an image region defined by a mask using commonly utilized basic metrics [42]. The gray-level dependence matrix (GLDM) quantifies the characteristics of gray-level dependency in an image, specifically the similarity or dependency of pixel values on their neighboring pixels. This feature can reveal the textural coarseness of tumor tissue [42]. Tumors with coarser textures might indicate greater tissue heterogeneity, which is sometimes associated with poorer treatment response and prognosis. The gray level co-occurrence matrix (GLCM) provides information on image texture, such as contrast and uniformity, related to the arrangement and structural heterogeneity of tumor cells. Similar to GLDM, these textural features may correlate with the biological behavior and prognosis of the tumor. The gray level size zone matrix (GLSZM) is highly relevant to tumor shrinkage and can quantify textural complexity, implying more complex biological characteristics of the tumor. [43, 44]. GLSZM, GLRLM, and GLCM are regional textural features and have been applied to emphasize local heterogeneity information, as the ability to distinguish patients with distinct prognoses has already been confirmed for other tumors [45–47]. A previous study demonstrated that heterogeneity in MRI distribution within tumors serves as a valuable biomarker for predicting treatment outcomes in patients with NPC [28]. A study also demonstrated that features such as GLCM, GLDM, and GLSZM extracted from PET-CT can predict local recurrence-free survival (LRFS) in nasopharyngeal carcinoma patients. Our study corroborates these findings, showing that these features can predict early rapid metastasis occurring two years posttreatment.

Previous research has demonstrated that these clinical factors might act as significant prognostic indicators for NPC patients [19], although they only had a modest predictive value for enhancing reclassification performance in this study. Lymphocytes play a major role in the immunological response of the host to tumors. Neutrophil granulocytes play an important role in controlling the circulatory angiogenesis of chemokines, growth factors, and proteases [19, 48]. Thus, we retained them as conceivable elements in the nomogram [49]. Previous studies have investigated the relationship between blood inflammation indicators and NPC prognosis, and an NLR ≤ 2.695 is associated with poor OS [19]. However, one study suggested that patients with an NLR ≥ 3 had worse survival [20]. In this study, the NLR was included in the nomogram model, and an NLR ≥ 3.8 indicated a greater probability of metastasis. This may be related to the diversity of the selected patients. The two different results may suggest a deeper mechanism waiting to be explored.

Our study has several limitations. First, due to the retrospective nature of the study, selection bias may be inevitable. The research sample size in this study was relatively small, and the data were derived solely from a single center. To address potential biases inherent in retrospective data analysis, a model was constructed and subsequently internally and externally validated through multicenter collaborations. However, the validation results may have been compromised due to the limited number of patients in the validation group. Therefore, future prospective studies encompassing larger populations, along with further external validation, are warranted to validate the conclusions drawn from this research. Additionally, it is worth noting that different MRI equipment was utilized for inspections, and the scanning parameters were not standardized. Future endeavors should focus on standardizing imaging protocols. Third, our study revealed the good predictive value of radiomic features, but the present study was limited because pretreatment did not include posttreatment or dynamic characteristics. Therefore, a prediction model that further integrates longitudinal data and images may improve the prediction power.

## Conclusion

Our study is the first to incorporate clinical features, hematological markers, and radiomic features, and our nomogram model seems to be an effective predictor of NPC outcomes. To improve the prognosis of NPC patients via layered care, a nomogram based on the NLR, T classification, N classification and radiomics for predicting early rapid metastasis in NPC patients may serve as a therapeutic personalized tool. Further research is also required to explore the generalized utility of our model and to translate it into clinical use.

### Supplementary Information

**Supplementary Material 1.**

**Supplementary Material 2.**

## Data Availability

The corresponding author will provide the datasets used and analyzed during the current work upon reasonable request.
