# Supplementary material for: Pretreatment multiparametric MRI radiomics-integrated clinical hematological biomarkers can predict early rapid metastasis in patients with nasopharyngeal carcinoma
Source: BMC Cancer. 2024 Apr 8;24:435. doi: 10.1186/s12885-024-12209-6 (PMC11003025; doi:10.1186/s12885-024-12209-6)

Supplementary Figure The feature screening processes of T1WI, CE-T1WI T2WI, and T2WI/FS, respectively. (A1, B1, C1, D1) Lasso coefficient profile plots show that the variations in the size of the coefficients of parameters shrink with an increasing value of the k penalty. (A2, B2, C2, D2) Penalty plot for the Lasso model with error bars denoting the standard errors. (A3, B3, C3, D3) Box plots of the non- and metastasis groups are subdivided based on the Rad score. (A4, B4, C4, D4) The AUC of Rad-score.


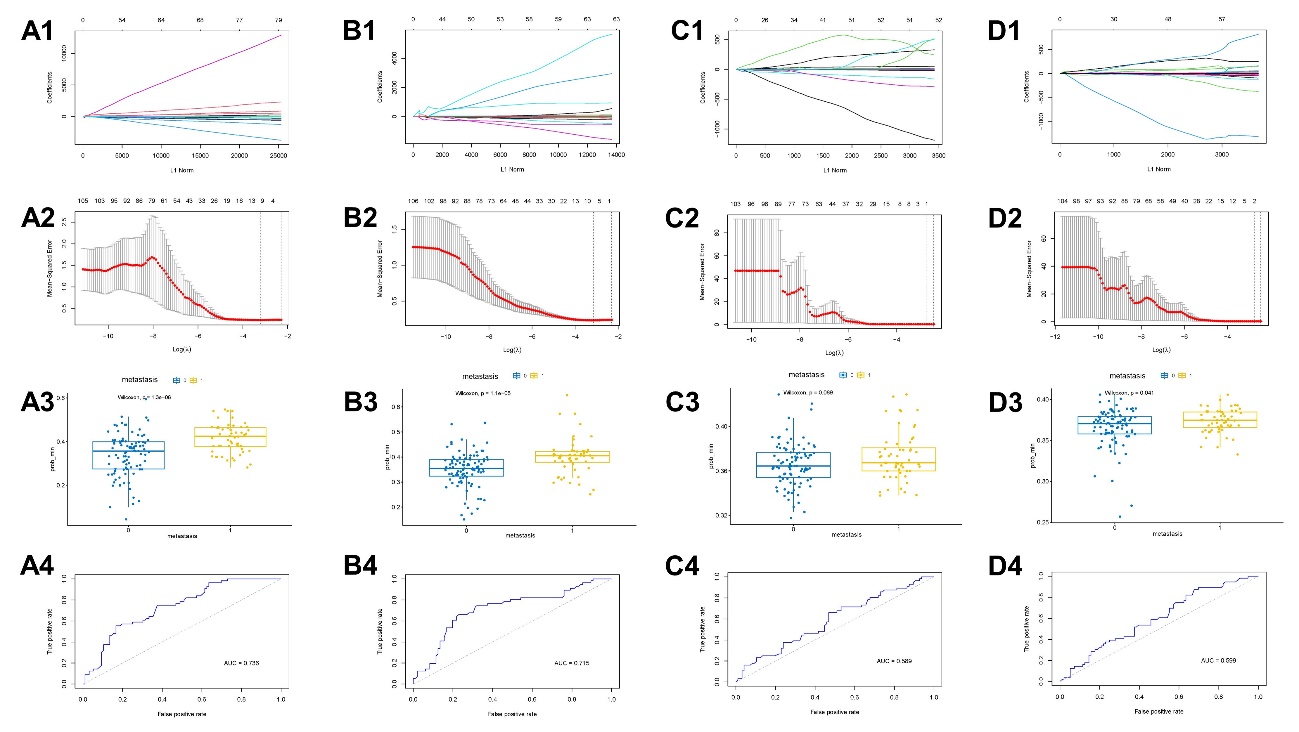

Supplement: Supplementary file 1 — Supplementary Material 1. [file 12885_2024_12209_MOESM1_ESM.docx]
