# Supplementary material for: Pretreatment multiparametric MRI radiomics-integrated clinical hematological biomarkers can predict early rapid metastasis in patients with nasopharyngeal carcinoma
Source: BMC Cancer. 2024 Apr 8;24:435. doi: 10.1186/s12885-024-12209-6 (PMC11003025; doi:10.1186/s12885-024-12209-6)
Supplement: Supplementary file 2 — Supplementary Material 2. [file 12885_2024_12209_MOESM2_ESM.docx]

**Supplementary table**

Pairwise comparison of ROC curves by Delong's test in training and validation dataset

|  | Clinical vs. radiomics | clinical vs. clinical+radiomics | radiomics vs. clinical+radiomics |
| --- | --- | --- | --- |
| Training dataset | 0.0472 | 0.0109 | ＜0.0001 |
| Validation dataset | 0.5336 | 0.2557 | 0.0564 |
